# A Coverage Optimization Method for WSNs Based on the Improved Weed Algorithm

**DOI:** 10.3390/s21175869

**Published:** 2021-08-31

**Authors:** Fang Zhu, Wenhao Wang

**Affiliations:** School of Computer and Communication Engineering, Northeastern University at Qinhuangdao, Qinhuangdao 066004, China; zhufang@neuq.edu.cn

**Keywords:** wireless sensor networks, coverage optimization, Levy flight, random walk, differential evolution

## Abstract

Wireless sensor networks (WSNs) is a multi-hop wireless network composed of a group of static or mobile sensor nodes in the form of self-organization. Uneven distribution of nodes often leads to the problem of over coverage and incomplete coverage of monitoring areas. To solve this problem, this paper establishes a network coverage optimization model and proposes a coverage optimization method based on an improved hybrid strategy weed algorithm (LRDE_IWO). The improvement of the weed algorithm includes three steps. Firstly, the standard deviation of normal distribution based on the tangent function is used as the seed’s new step size in the seed diffusion stage to balance the ability of the global search and local search of weed algorithm. Secondly, to avoid the problem of premature convergence, a disturbance mechanism combining enhanced Levy flight and the adaptive random walk strategy is proposed in the process of seed breeding. Finally, in competition of invasive weed stage, the differential evolution strategy is introduced to optimize the competition operation process and speed up convergence. The improved weed algorithm is applied to coverage optimization of WSNs. The simulation results show that the coverage rate of LRDE_IWO is increased by about 1% to 6% compared with the original invade weed algorithm (IWO) and the differential evolution invasive weed optimization algorithm (DE_IWO), and the coverage rate of the LRDE_IWO algorithm is increased by 4.10%, 2.73% and 1.19%, respectively, compared with the antlion optimization algorithm (ALO), the fruit fly optimization algorithm (FOA) and the gauss mutation weed algorithm (IIWO). The results prove the superiority and validity of the improved weed algorithm for coverage optimization of wireless sensor networks.

## 1. Introduction

With the development of wireless communication technology, wireless sensor networks (WSNs) have been widely used in many fields of social life because of its advantages of low-power consumption, low cost, wide coverage and diversified integration functions [1]. WSNs is an intelligent self-organizing network composed of multiple sensor nodes with limited computing, storage and wireless communication capabilities. In order to achieve full coverage of the monitoring area, a large number of nodes are usually deployed, which is not only costly but also can lead to communication conflicts. Therefore, how to optimize the deployment of nodes, that is, to cover a larger area with fewer nodes, has become a hotspot in the research of WSNs [2,3].

In general, there are two types of network coverage optimization methods. Firstly, there is coverage optimization based on geometric methods. For example, the geometric coverage scheme based on arc area algorithm (GCAS) proposed in Wang et al. [4] is optimized by combining genetic algorithms with geometric relationships between nodes; although the GCAS strategy has high coverage and high robustness, it still cannot achieve 100% coverage. Mu et al. [5] proposed a bee colony algorithm optimized with Voronoi (VABC) to guide the deployment of mobile nodes, which improves the coverage ratio of the network and achieves the optimal coverage of the network. However, VABC has a “premature” problem. Secondly, there is the application of the intelligent optimization algorithm to complete dynamic coverage optimization of node deployment. In recent years, some researchers have used swarm intelligence algorithms to solve the problem of WSN coverage, for example, particle swarm optimization algorithm (PSO), whale optimization algorithm (WOA), gray wolf optimization algorithm (GWO), artificial fish swarm algorithm (AFSA), etc. Due to the advantages of few parameter settings, simple calculation and easy-to-parallel processing, they are applied in WSN coverage optimization [6,7,8,9]. Among them, the ant lion algorithm (ALO) is applied in WSN coverage optimization in [10], which acquires good results, but is prone to fall into local optimum problems. In [11], the improved strategy of the fruit fly optimization algorithm (FOA) is introduced, which greatly improves the network coverage. However, it has the problem of unwanted rapid convergence that results in low coverage accuracy. One paper [12] proposed an invasive weed algorithm with Gauss mutation (IIWO) for WSN coverage optimization. Compared with the original weed algorithm, this algorithm can enhance the diversity of population and improve network coverage but easily fall in the “early-maturing phenomenon”. An improved grey wolf optimization algorithm is presented in [13], which has improved WSN coverage optimization application and network coverage. However, the improved algorithm converges slowly in the early stage that leads to unstable coverage. In [14], by introducing hyperbolic sine regulatory factor, dynamic cosine wave weight coefficient and mutation strategy based on Laplacian and Gaussian distribution, a higher network coverage is obtained. Moreover, the problem of WSN coverage can be solved by also using Cluster-Heads (CHs) that relocate themselves inside clusters in such a way that the total energy consumption in the network is reduced and the network lifetime is extended, (e.g., [15]). In this paper, the problem of WSN coverage refers to analyzing the deployment location of the optimized nodes through simulation before deploying the nodes, and finally deploying each node manually or by using robots. Therefore, this paper does not consider the energy consumption of the nodes.

The invasive weed optimization algorithm (IWO) is a new meta-heuristic optimization algorithm proposed by Mehrabian AR, which is widely used in multi-objective optimization problems [16], but rarely applied in WSN node deployment optimization. Like other algorithms, the basic IWO algorithm is prone to premature convergence and low convergence accuracy. One paper [12] has achieved good results in optimizing WSN coverage. Therefore, inspired by [12], this paper proposes an improved weed algorithm based on a hybrid strategy (LRDE_IWO), to solve the problem of slow convergence speed of the IWO algorithm and to ensure it easily falls into the local optimum, it can significantly improve the coverage rate of WSN. The contributions of this paper are mainly reflected in the following three aspects:

1. A standard deviation of the tangent function is designed to replace the original standard deviation. The value in the early iteration changes relatively quickly, which is conductive to a global search. In the latter part of the iteration, the value changes relatively slowly and the step size of the weed movement is small, which is beneficial to a local search. Obviously, this strategy can balance the global search and local development ability of the algorithm, and the convergence speed of the algorithm is improved.
2. In this paper, the diversity of the population is increased by adding the disturbance mechanism of enhanced Levy flight and the adaptive random walk strategy.
3. In order to further improve the convergence ability of the algorithm and avoid local optimization, this paper introduces the differential evolution strategy. Selecting a certain number of excellent weed seeds for differential evolution can save more elite individual weeds and improve the coverage rate of WSN.

The standard deviation of the tangent function strategy is utilized to improve the convergence speed of the algorithm, the disturbance strategy of enhanced Levy flight and the adaptive random walk strategy is used to expand the diversity of the weeds and the differential evolution strategy is used to enhance the ability of the algorithm to jump out of the local optimum.

The rest of this paper is organized as follows. The section “WSN Node Coverage Model” builds the WSN coverage model. The sections “Standard Invasive Weed Algorithm” and “Improved Weed Algorithm Based on Hybrid Strategy” describe the improved weed algorithm in detail. In the section “WSN Coverage Optimization”, the steps and algorithm flow of how the improved weed algorithm is applied to WSN node deployment optimization are described in detail. The “Simulation and Analysis” section discusses the experimental results of the coverage optimization method (LRDE_IWO) compared with other algorithms. The work of this paper is summarized in the “Conclusion” section.

## 2. WSN Coverage Model

There are mainly two types of WSN coverage models [17]: the Boolean coverage model and the Probability coverage model. In this paper, we use the same probabilistic coverage model as [18].

Assume that N sensor nodes randomly deployed in WSN are isomorphic, and the sensing radius and communication radius are $R_{s}$ and $R_{c}$, respectively. To ensure the connectivity of the WSN, the communication radius of the node is set to be greater or equal to twice the sensing radius of the node. Suppose that the set of wireless sensor nodes is $V=\{v_{1},v_{2},\cdots ,v_{N}\}$, the coordinates of *v_i_* is $\left(x_{i},y_{i}\right)$. For the convenience of calculation, the rectangular area is discretized as m×n grid points to be covered, while the set of grid points is $u_{j}=(x_{j},y_{j}),j\in \{1,2,\cdots ,m\times n\}$.

If the distance between the grid point $u_{j}$ and one of the nodes is less than or equal to the sensing radius $R_{s}$, the grid point is considered completely covered by WSN. The distance between node $v_{i}$ and grid point $u_{j}$ is defined as Formula (1):

(1) $$d(v_{i},u_{j})=\sqrt{{\left(x_{i}-x_{j}\right)}^{2}+{\left(y_{i}-y_{j}\right)}^{2}}$$

The probability $p(v_{i},u_{j})$ that the grid point $u_{j}$ is monitored by the node $v_{i}$ is defined as Formula (2):

(2) $$p(v_{i},u_{j})=\{\begin{array}{l} 0,R_{s}\leq d(v_{i},u_{j})\\ \exp(\Delta ),R_{s}-R_{e}<d(v_{i},u_{j})<R_{s}\\ 1,R_{s}-R_{e}\geq d(v_{i},u_{j}) \end{array}$$

where $\Delta =-\lambda \frac{d(v_{i},u_{j})-R_{s}-R_{e}}{R_{s}-d(v_{i},u_{j})}$, $R_{e}$ is the sensing error radius and λ is the sensing attenuation coefficient. 

Then, the joint sensing probability of all sensor nodes to grid point $u_{j}$ is described as Formula (3):

(3) $$p(V,u_{j})=1-\prod_{i=1}^{N}\left[1-p(v_{i},u_{j})\right]$$

where V is the set of all sensor nodes within the monitoring region. The rate of coverage is defined as the ratio that the total perceived probability of grid points covered by the node set V to the total number of grid points in the area. The coverage rate $R_{cov}$ can be expressed as Formula (4):

(4) $$R_{cov}=\frac{\sum_{j=1}^{m\times n}p(V,u_{j})}{m\times n}$$

From Formula (4), we can see that the problem of coverage is transformed into finding the optimal solution of $R_{cov}$. In our coverage optimization method, we applied the improved weed algorithm to obtain the optimal value of $R_{cov}$.

***Notes:***

**1.** In the Boolean covering model, p=1 when the grid points are covered, otherwise p=0; in the probability coverage model, p=1 when the grid points are covered, otherwise p is a value between 0 and 1 or 0, which depends on the formula of the probability model.

**2.** The reason why the area coverage is calculated by dividing the grid points is that it is difficult to implement in MATLAB directly according to the ratio of the area covered to the total area of the monitoring region. Thus, this strategy is adopted in this paper, i.e., dividing the grid points and calculating the distance between each node and the grid point. If the distance is less than the perceived radius, the grid point is covered, otherwise, coverage probability is calculated according to the formula for probability coverage model. Finally, the area coverage is the ratio of the sum of coverage probabilities of all grid points of the total number of grid points.

## 3. Standard Invasive Weed Algorithm

The invasive weed algorithm simulates the basic process of weed diffusion, growth, reproduction and competitive extinction in the natural environment. The algorithm mainly consists of five steps, namely population initialization, growth and reproduction, spatial diffusion, competitive survival and stop criteria [19]. In the coverage optimization method, Formula (4) acts as an objective function value. Each individual weed is as an objective function value, namely the coverage of WSN. The specific steps are as follows.

(1) Initialize the population. The position of the *i*-th weed is randomly generated by the following Formula (5):

(5) $$X_{i}=X_{L}+\mathrm{rand}(0,1)\cdot (X_{U}-X_{L})$$

where $X_{i}=(x_{i1},x_{i2},\cdots ,x_{iD})(i\in \{1,2,\cdots ,P_{ini}\})$, *D* is the dimension of the solution vector, $P_{ini}$ is the initial population size and $X_{U}$ and $X_{L}$ are the upper and lower bounds of the value of $X_{i}$, respectively.

(2) Growth and reproduction. Calculate the fitness value $f(X_{i})$ for each individual weed, that is, the coverage of WSNs. Each individual generates more seeds based on the maximum and minimum fitness values $f_{\max}$ and $f_{\min}$ in the population, and the individuals with the better population (i.e., the better fitness values) produce more seeds. The number of seeds produced by each individual can be calculated by Formula (6):

(6) $$S=\left\lfloor S_{\min}+\frac{f(X_{i})-f_{\min}}{f_{\max}-f_{\min}}(S_{\max}-S_{\min})\right\rfloor$$

where $S_{\min}$ and $S_{\max}$ are the minimum and maximum number of seeds, respectively, and ⌊•⌋ shows rounding down.

(3) Spatial diffusion. Seeds produced by weeds are randomly distributed in the neighborhood of parental individual in the form of normal distribution $N(0,\sigma^{2})$. In the evolution of the algorithm, the standard deviation is calculated by Formula (7):

(7) $$\sigma_{t}=\sigma_{final}+{\left(\frac{T-t}{T}\right)}^{w}(\sigma_{ini}-\sigma_{final})$$

where $\sigma_{t}$ is the standard deviation for the *t*-th iteration, $\sigma_{ini}$ and $\sigma_{final}$ is the initial and final standard deviation for sowing seeds, respectively, w is a non-linear regulatory factor with a general value of w=3 and T is the maximum number of iterations. With the number of iterations increasing, seeds are gradually distributed around the paternal individuals, which makes the transition from global exploration to local development to improve the algorithm’s search speed.

R-selection means “live fast, reproduce quick, die young” and K-selection means “live slow, reproduce slow, die old” [20]. Formula 6 is the stage of weed growth and reproduction, focusing on R-selection; Formula 7 is the seed diffusion stage, focusing on K-selection. The physical meaning of Formula 6 and Formula 7 reflects the transformation of weeds from R-selection to K-selection.

(4) Competitive survival. After several iterations, when the number of populations exceeds the maximum population size $P_{\max}$, all individuals are sorted according to the fitness values, excluding those with poor fitness and retaining the rest; that is, the algorithm first reproduces rapidly through the weeds, occupying the living space, and then keeps the more competitive weeds to continue the spatial search.

(5) Stop criteria. Repeat steps (2) to (4) to record the individuals with the best fitness value in each generation until the maximum number of iterations T is reached, the algorithm stops and the global optimal solution is output.

## 4. Improved Invasive Weed Algorithm Based on the Hybrid Strategy

### 4.1. Standard Deviation Strategy of Normal Distribution Based on the Tangent Function

In the weed algorithm’s diffusion stage, the algorithm’s convergence speed and solution accuracy are closely related to the size of the non-linear adjustment factor w. When w is larger, the algorithm converges quickly but the solution accuracy is low, and when w is smaller, the solution accuracy is high but convergence is slow, and it is easy to fall into the local optimum. Therefore, inspired by [21], the original standard deviation formula is transformed by the tangent function. The following Formula (8) is derived from the features of the tangent function:

(8) $$\sigma =\tan\left(0.875\times \frac{T-t}{T}\right)\times (\sigma_{ini}-\sigma_{final})+\sigma_{final}$$

**Figure 1:**
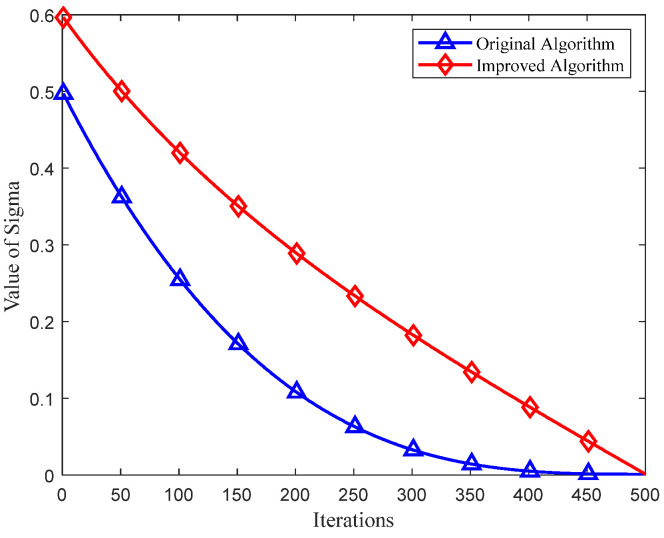
Comparing the standard deviation of the improved weed algorithm with that of the original algorithm.

In Figure 1, σ is nonlinearly decreasing from 0.6 to 0. The value of σ in the early iteration changes relatively quickly, which is conductive to global search. In the latter part of the iteration, the value of changes relatively slowly and the step size of weed movement is small, which is beneficial to local search. Obviously, this strategy can balance the global search and local development ability of the algorithm, and the convergence speed of the algorithm is improved. 

### 4.2. Enhanced Levy Flight and the Adaptive Random Walk Strategy

In the stage of seed breeding, in order to avoid the algorithm falling into the local optimum, all individual weeds are selected after step (3), and the enhanced Levy flight or adaptive random walk strategy is used to mutate them with a certain probability. Then, the individuals with better fitness values are greedily selected as the current individual weeds. The Levy flight strategy is used for global exploration, so that individuals are widely distributed in the search space to improve the ability of global optimization. The random walk strategy is used to optimize the individual weeds in a concentrated area to improve the local search ability [22].

**Figure 2:**
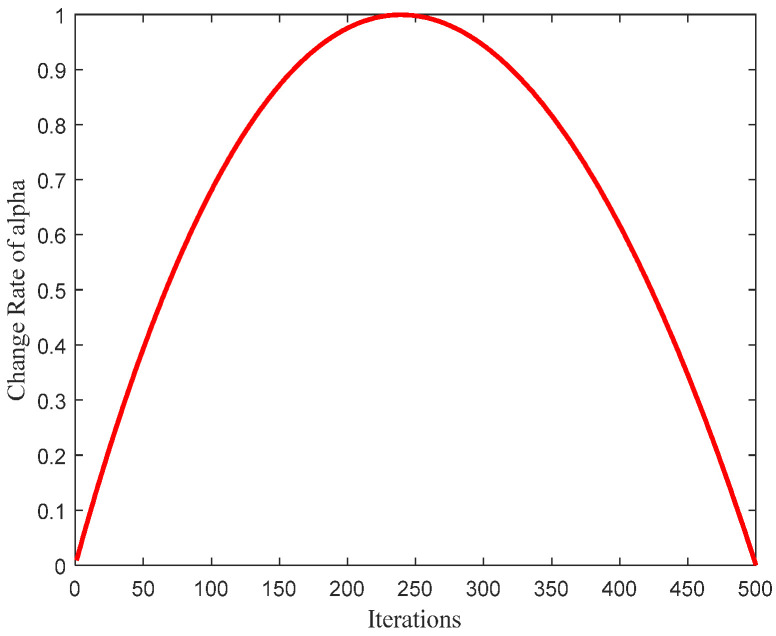
Change of step size factor α.

Although the basic Levy flight mechanism increases the global search ability of the algorithm in the optimization process, it also has the problem of falling into the local optimum. An enhanced Levy flight strategy with adaptive step factor α is proposed, which changes step α from a fixed value to a dynamic step factor that changes with the number of iterations. The formula for calculating the dynamic step factor α is as follows:

(9) $$\alpha (t)=\frac{t}{T}\sinh\left(1-\frac{t}{T}\right)\cdot R$$

where t is the current number of iterations, T is the maximum number of iterations and R is the tune parameter. After several experiments, the tune parameter R=3.83 can meet the α(t)∈[0,1] condition and enhance Levy flight to find the optimal value [23]. Figure 2 shows a change rate of factor α with iterations.

Levy flight [24] obeys the Levy distribution. The mathematical model of the mutated Levy flight mechanism is shown in Formulas (11) and (12), and Formula (10) is the position updating formula:

(10) $$x_{i}^{t+1}=x_{i}^{t}+l⊕Levy(\lambda )$$

where $x_{i}^{t}$ denotes the current position of individual weeds, $x_{i}^{t+1}$ denotes the position of the next generation of individual weeds, ⊕ denotes point-to-point multiplication, l represents the weight of the control step and $l=\alpha (t)\cdot (x_{i}^{t}-x_{best})$, where $x_{best}$ is the current optimal location, and Levy(λ) represents a function that obeys the Levy distribution and satisfies $Levy\sim u=t^{-\lambda },1<\lambda \leq 3$. Levy(λ) can be calculated by Formula (11):

(11) $$Levy(\lambda )=\frac{\varphi \times \mu }{|\nu {|}^{\frac{1}{\lambda }}}$$

φ can be calculated with Formula (12):

(12) $$\varphi ={\left[\frac{\Gamma \left(1+\lambda \right)\times \sin\left(\pi \times \frac{\lambda }{2}\right)}{\Gamma \left(\frac{1+\lambda }{2}\right)\times \lambda \times 2^{\frac{\lambda -1}{2}}}\right]}^{\frac{1}{\lambda }}$$

where μ and ν follow normal distribution, λ is 1.5. The formulas are as follows:

(13) $$\mu \sim N(0,\sigma_{\mu }^{2}),\nu \sim N(0,\sigma_{\nu }^{2})$$

(14) $$\sigma_{\mu }={\left[\frac{\Gamma \left(1+\lambda \right)sin\left(\frac{\pi \lambda }{2}\right)}{\lambda \cdot \Gamma \left(\frac{\lambda +1}{2}\right)\cdot 2^{\frac{\lambda -1}{2}}}\right]}^{\frac{1}{\lambda }},\sigma_{\nu }=1$$

As shown in Figure 2, the Levy global search from subspace to global solution space is achieved by introducing a dynamic adjustment of the search step so that the change of the step factor is proportional to the number of iterations in the first half of the algorithm. At the later stage of the algorithm, the change of step factor gradually decreases, which leads to the weakening of global search and the enhancement of local development capability. This method strengthens Levy flight mechanism’s search for solution space and effectively balances the ability of global search and local development.

**Figure 3:**
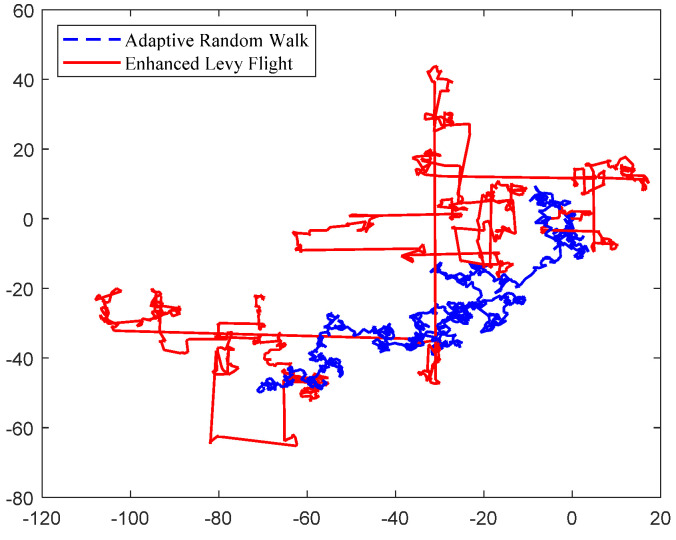
Simulation of enhanced Levy flight and the adaptive random walk strategy.

The random walk algorithm uses mixed variation and crossover to produce new solutions, which can enhance the diversity of the population, avoid the attraction of local optimum to some extent and improve the local search ability of the algorithm. Based on the improvement of random walk, an adaptive step size random walk strategy is proposed. The specific calculation is shown by Formula (15):

(15) $$\begin{array}{l} x_{i}^{t+1}=x_{i}^{t}+\varepsilon \left(t\right)\cdot \left(x_{j}(t)-x_{k}(t)\right)\\ \varepsilon (t)=\mathrm{rand}(0,1)\cdot \left[2-t/(T-1)\right] \end{array}$$

where $x_{j}^{t}$ and $x_{k}^{t}$ are the positions of two random individuals in the *t*-th iteration and rand(0,1) is a random number uniformly distributed among (0,1). Figure 3 shows the comparison of the simulation experiments of enhanced Levy flight and adaptive random walk strategy with 1000 iterations. It can be seen that enhanced Levy flight has a larger search range because of the special walking mode, which is more conducive to the global search of the optimization algorithm; the adaptive random walk strategy has a relatively concentrated search range, which is more conductive to local optimization in the development stage.

Although the above disturbance strategy can update the position, it cannot guarantee that the new solution is better than the original solution. Therefore, the greedy selection mechanism is used to compare the fitness of the original solution and the new solution to retain the better solution:

(16) $$x_{i}^{t}=\{\begin{array}{l} x_{i}^{t},\;f(x_{i}^{t})>f(x_{i}^{t+1})\\ x_{i}^{t+1},f(x_{i}^{t})<f(x_{i}^{t+1}) \end{array}$$

### 4.3. Differential Revolution Strategy

Differential evolution algorithm (DE) is a swarm intelligence optimization algorithm proposed by Storn and Price in 1995 [25]. It uses the differential information among individuals to disturb the individual vector, and evolves better offspring through mutation, crossover and selection [26]. In the stage of seed competitive survival, the differential evolution strategy is introduced to mutate individuals, and then the greedy selection principle is used to update the current individual position. In order to speed up the operation time of the algorithm, the first 40% of the optimal individuals are selected for differential evolution, and the process is described below.

(1) Differential mutation operation. Randomly select three different individuals $x_{r_{1}}^{t},x_{r_{2}}^{t},x_{r_{3}}^{t}$ from the parent (*t* iteration) and generate a new mutant individual based on the following Formula (17):

(17) $$v_{i}^{t}=x_{r_{3}}^{t}+F\cdot (x_{r_{1}}^{t}-x_{r_{2}}^{t})$$

where t is the current generation and the scaling factor F is a constant between [0,2], F=0.5.

(2) Crossover operation. Crossover is a binomial hybridization between the mutant $v_{i}^{t}$ and the parent $x_{i}^{t}$ in the differential mutation operation to produce the test individual $u_{i}^{t}$, in order to enhance the diversity of the population. The process is calculated as Formula (18):

(18) $$u_{i,j}^{t}=\{\begin{array}{l} v_{i,j}^{t},\;\mathrm{rand}(0,1)\leq CR\;or\;j=j_{rand}\\ x_{i,j}^{t},\;otherwise \end{array}$$

where j∈{1,2,⋯,D}, D is the dimension of the individual vector, $j_{rand}$ is a random integer between [1,D] and CR is the crossover probability.

(3) Selection operation. The selection operation of DE algorithm is a sort of elite screening mechanism. By using the greedy principle and calculating the fitness values of the target vector and the test vector, the individuals with better fitness values are selected as the individuals of the next generation population:

(19) $$x_{i}^{t+1}=\{\begin{array}{l} u_{i}^{t},\;f(u_{i}^{t})\geq f(x_{i}^{t})\\ x_{i}^{t},\;f(u_{i}^{t})<f(x_{i}^{t}) \end{array}$$

### 4.4. LRDE_IWO Algorithm Pseudo Code

In this paper, the weed algorithm’s initial population size is equal to the maximum population size, that is $\left|P_{ini}\right|=\left|P_{\max}\right|=N$. The specific process is shown in Algorithm 1.
**Algorithm 1** LRDE_IWO algorithm**Input**: Population size N, the maximum number of iterations T, the exploration iteration ratio φ and other parameters. **Output**: Global optimal solution $g_{best}$.   1. **for**
*t* = 1 to *T* **do**   2. Calculate the maximum fitness value of the population
   $f_{\max}$ and minimum fitness value $f_{\min}$   3. The standard deviation of this iteration is calculated        according to Equation (8)   4. **for** each individual weed **do**   5. Determine the number of seeds produced by each        weed according to Equation (6)   6. Seeds produced by random allocation   7. The fitness of seeds was calculated   8. if rand<0.5−0.5t/(φ∗T) then   9. Update the individual position according to     Equation (10)  10. **else**  11. Update the individual position according to         Equation (15)  12. **end if**  13. Greedily select the current optimal individual        according to the fitness value  14. **end for**  15. The populations were merged and sorted by fitness       value  16. The top 40% of the excellent individuals were selected        for differential evolution according to Formulas (16)–(18)  17. Greedy selection of the above individuals according to        fitness values  18. Update global optimal solution $g_{best}$**end for**

### 4.5. Algorithm Complexity Analysis

Suppose *m* is the maximum number of iterations of the algorithm, *n* is the population size, *d* is the dimension of the optimization problem and *f*_min_ and *f*_max_ are the minimum and maximum number of the seeds, respectively. The LRDE_IWO algorithm first initializes the population, and its time complexity is O(*nd*). Firstly, the fitness is calculated according to the position of the weed and disturbance strategy, while the time complexity is O(*n*). Then, the seeds are created. The time complexity is *O*(*n*(*f*_max_ − *f*_min_)). Secondly, the weeds are sorted in ascending order according to the value of the fitness. The time complexity is O(*nlogn*). Finally, the position of the weed population is updated and selected according to the differential revolution strategy, and the time complexity is O(*n*). Thus, the total time complexity is *O*(*m*(*nd* + *n* + *n*(*f*_max_ − *f*_min_) + *nlogn* + *n*)), that is O(*mn*). Compared to IWO, LRDE_IWO adds mutation steps, but still belongs to the operation of weed update location, without adding additional time complexity. Thus, the total time complexity of LRDE_IWO is the same as that of IWO.

## 5. WSN Coverage Optimization

The target of WSN coverage optimization based on the LRDE_IWO algorithm is as follows. A certain number of sensor nodes are used to optimize the location where they will be deployed, in order to maximize the coverage of the target monitoring area. In other words, the optimal locations of these nodes are obtained to maximize $R_{\mathrm{cov}}$. The coverage optimization problem is abstracted as a high-dimensional vector optimization problem with $R_{\mathrm{cov}}$ as the objective function. The process of node location optimization in coverage optimization problem is abstracted as the behaviours of weed cloning, occupation, growth and reproduction, and the optimal solution is to deploy the target location of each node. In the algorithm, each individual weed represents a coverage distribution. If the nodes’ number is N, the individual dimension is 2N, in which 2i-1-th and 2i-th dimension values represent the abscissa and ordinate of the node, respectively. The specific algorithm steps are as follows.

**Step 1:** Input nodes’ number V, sensing radius *R_s_*, area side length L, etc., and the population size N of the algorithm is set to 30 and the maximum iteration number T is 300.

**Step 2:** Random initialization of the population position of LRDE_IWO.

**Step 3:** Using coverage $R_{\mathrm{cov}}$ as the objective function to calculate individual fitness values and record global optimal solution $g_{best}$.

**Step 4:** Calculate the improved standard deviation based on Equation (8) and the number of seeds produced based on Equation (6) by each individual.

**Step 5:** Select either enhanced Levy flight or the adaptive random walk strategy to mutate each individual according to Equation (10) or Equation (15). Then, greedily select an individual with higher fitness values than the current individual.

**Step 6:** Disturbance updates using the differential evolution of Equations (16)–(18) for the position of the first 40% of the best individuals. Compared with the original position, the better position is retained; otherwise, the disturbed position is discarded.

**Step 7:** Update global solution $g_{best}$.

**Step 8:** Determine whether the algorithm has reached the maximum number of iterations, and if so, outputs the global optimal solution, that is, the optimal coverage and the location coordinates of the corresponding node deployment. Otherwise, skip to Step 3 for the next iteration. 

**Chart 1:**
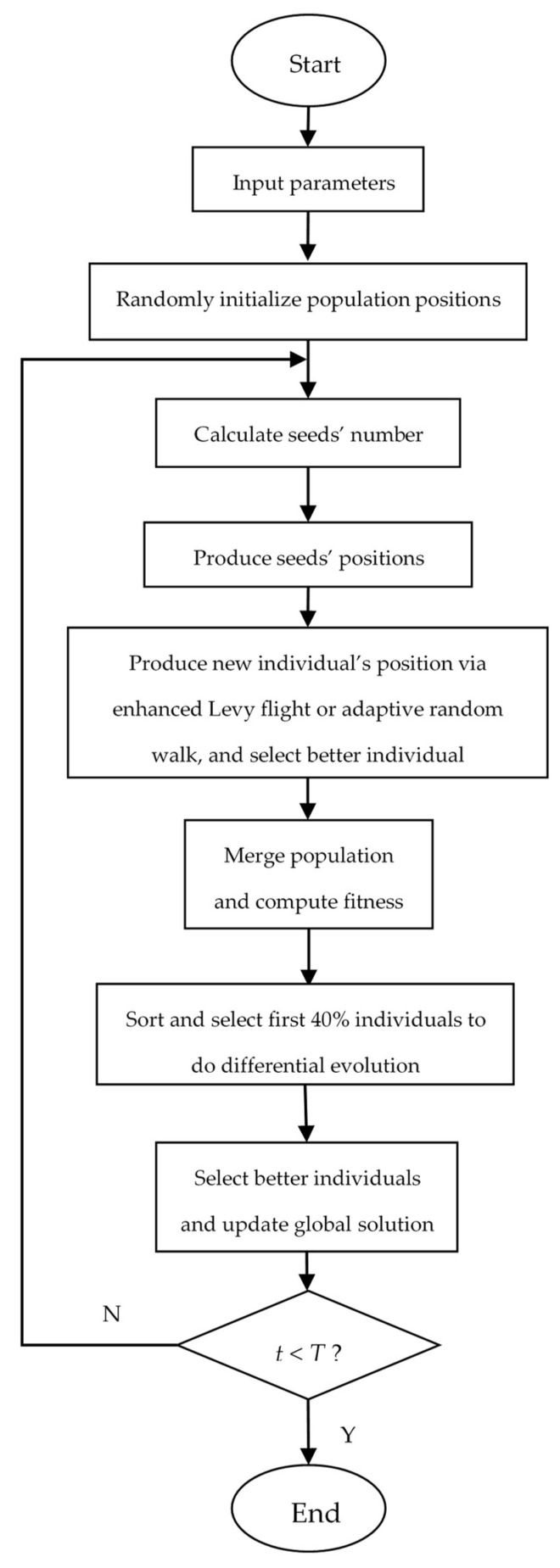
Flowchart of the LRDE_IWO algorithm.

The flowchart of the LRDE_IWO algorithm applied to the coverage optimization of WSN is shown in Chart 1 below.

## 6. Simulation Experiments and Analysis 

### 6.1. Algorithmic Performance Indicators

We assume that the boundary is outside the middle square area of [0.1L,0.9L]×[0.1L,0.9L], where *L* is the side length of the monitoring area. The boundary coverage and area coverage are calculated in the same way, the main difference being that the area in which the grid points are divided is different, the area coverage is divided into grid points within the entire monitoring area and the boundary coverage is divided into grid points within the boundary area.

***Notes:***

**1.** The area coverage is the coverage of all nodes within the monitoring range; the boundary coverage is the coverage of all nodes in the boundary range.

**2.** Because the coverage hole often occurs at the edge of the region, this paper proposes the concept of boundary coverage, that is, assuming that the small area outside the square area of [0.1L,0.9L]×[0.1L,0.9L] is defined as the boundary range, and the boundary coverage is calculated according to the probability model within this range, so as to quantify the coverage effect of the regional boundary.

### 6.2. Experimental Environment and Parameter Setting

In order to verify the performance of WSN coverage optimization method with the LRDE_IWO algorithm, two experiments were performed. Experiment 1 compares the LRDE_IWO algorithm with DE_IWO [27] and the original IWO algorithm. Experiment 2 compares the coverage effect of LRDE_IWO with ALO, FOA and IIWO. In the experiments, the parameters are set as the same as [10,11,12]. The experiment is carried out using a computer with an Intel core i7 dual-core CPU, clocked at 3.6 GHz, 8 GB of memory, with Windows 10 environment and using the experimental simulation software in MATLAB 2018a.

In this paper, the parameters values are as follows. In the WSN coverage model, $R_{c}=2R_{s},R_{e}=0.1R_{c},\lambda =0.1$. In the LRDE_IWO algorithm, the minimum and maximum number of seeds $S_{\min}=0$ and $S_{\max}=5$, the initial and final standard deviation $\sigma_{ini}=0.5$ and $\sigma_{final}=0.001$ and the exploration iteration ratio φ=0.95.

### 6.3. Experimental Results

#### 6.3.1. Compared with the Other Algorithms Based on IWO

The IWO algorithm, DE_IWO algorithm and LRDE_IWO algorithm are simulated. The algorithms were run 20 times independently, with 300 iterations each time. The experimental parameter setting (unit: m) is shown in Table 1.

The experimental data are shown in Table 2, Table 3 and Table 4. The “cr” in all the following tables represent the coverage rate.

**Figure 4:**
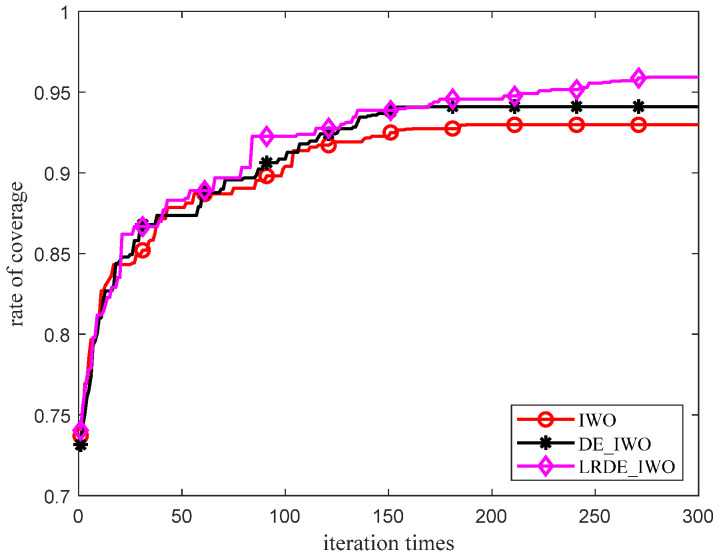
The iterative curves of the three algorithms.

From Table 2, Table 3 and Table 4, it can be seen that the optimized coverage rate of the LRDE_IWO algorithm is better than that of DE_IWO and IWO, and the standard deviation is smaller, which indicates that the optimized result of LRDE_IWO algorithm can be stably maintained near the average coverage rate. Taking the setting in Table 2 as an example, Figure 4 shows the optimization iteration curve of the three algorithms. From this figure, it can be concluded that the coverage rate of each generation of LRDE_IWO algorithm is higher than the other two algorithms. When other algorithms converge, LRDE_IWO can continue to search for optimization. It indicates that LRDE_IWO algorithm can jump out of the local optimal solution and improve the coverage accuracy.

**Figure 5:**
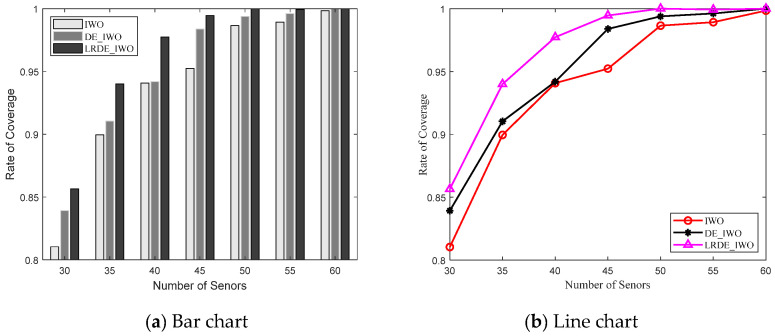
Bar chart and line chart of coverage varying with the number of nodes.

In order to explore the influence of the number of sensor nodes on the coverage rate, a two-dimensional plane with 50 m × 50 m monitoring area is set, and its sensing radius is $R_{s}=5$ m. Figure 5 shows the tendency of coverage with the number of nodes under different algorithms, in which (a) shows that the bar chart of coverage with nodes’ number, and (b) shows that the line chart of coverage with nodes’ number.

As shown from Figure 5, when the number of nodes is determined, the coverage rate of the LRDE_IWO algorithm is always better than other algorithms, and reaches 100% when there are 50 nodes. The other two algorithms barely approach or reach 100% coverage when there are 60 nodes. That shows that the LRDE_IWO algorithm can effectively coordinate the global exploration and local development ability of the algorithm, and thus, improve the effective coverage rate of the WSN nodes.

Taking the setting in Table 2 as an example, the average boundary coverage of LRDE_IWO, DE_IWO and IWO are 95.64%, 93.67% and 93.16%, respectively. This is because the three strategies of LRDE_IWO can effectively balance the ability of global search and local development and improve the probability of jumping out of local optimum to reduce the coverage blind area quickly.

#### 6.3.2. Compared with the Coverage Optimization Algorithms Based on ALO, FOA and IIWO

##### (1) Compared with the Coverage Optimization Algorithm Based on ALO

**Figure 6:**
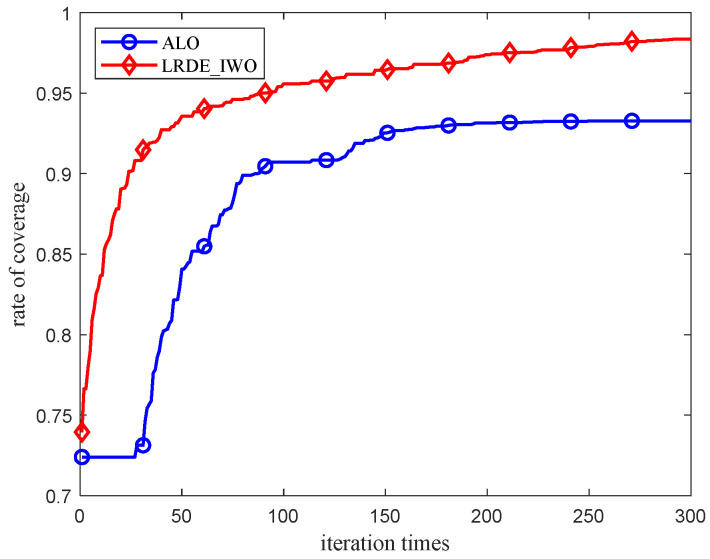
ALO and LRDE_IWO coverage optimization iterative curve.

**Figure 7:**
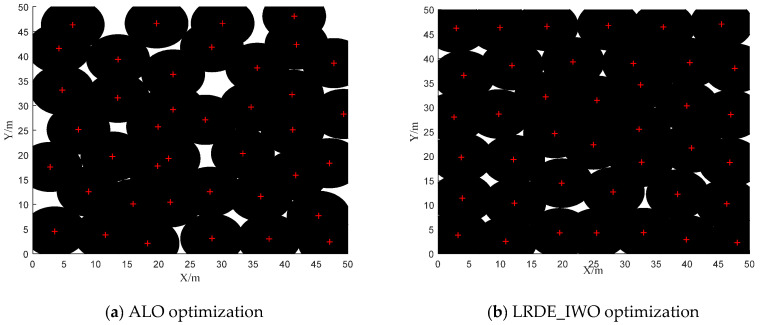
Node distribution optimized by ALO and LRDE_IWO.

The experimental parameters are set as follows: the monitoring area is a two-dimensional plane of 50 m × 50 m, which is discretized into 51 × 51 grid points, the number of sensor nodes is *N* = 40, the sensing radius is $R_{s}=5$ m and the communication radius is $R_{c}=10$ m, and it runs 20 times independently with 300 iterations each time. Figure 6 shows the iteration curve of the ALO algorithm and LRDE_IWO algorithm in coverage optimization, Figure 7 shows the node deployment diagram after optimization and Table 5 shows the comparison of the ALO and LRDE_IWO optimization coverage results. 

It can be seen from Table 5 that after node deployment optimizations for 20 times in this area, the average, best and worst coverage rate obtained by LRDE_IWO has increased by 4.10%, 2.51% and 4.50%, respectively, compared with the ALO algorithm. Moreover, the standard deviation of the coverage is relatively smaller, which indicates that the optimized coverage rate can be maintained at more than 96.43%. It can be seen from Figure 6 that the algorithm has faster convergence speed and higher coverage accuracy than the ALO algorithm. It can be seen from Figure 7 that the LRDE_IWO algorithm achieves approximately complete coverage of the area.

It is obvious from Figure 7 that (b) covers the boundary more closely than (a). The simulation results show that the boundary average coverage of (b) is 96.73%, while that of (a) is 85.07%. This is because the improved algorithm can balance the global search and local search well, and can jump out of the local optimum.

##### (2) Compared with the Coverage Optimization Algorithm Based on FOA

**Figure 8:**
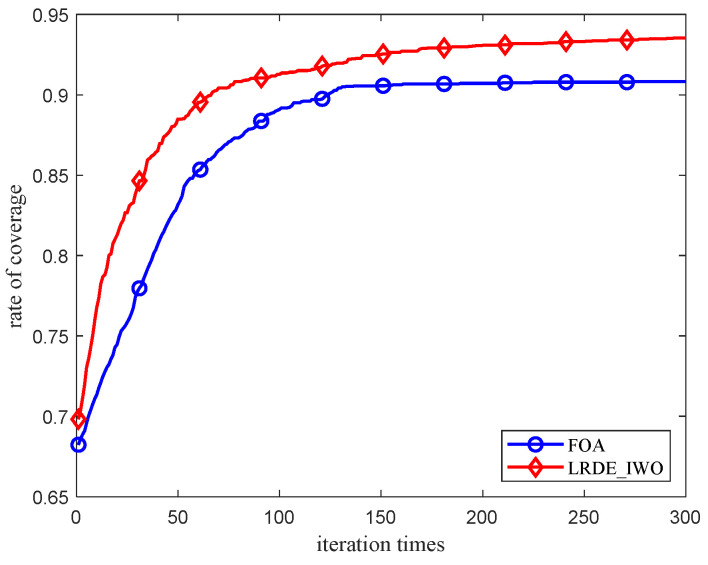
FOA and LRDE_IWO coverage optimization iterative curve.

**Figure 9:**
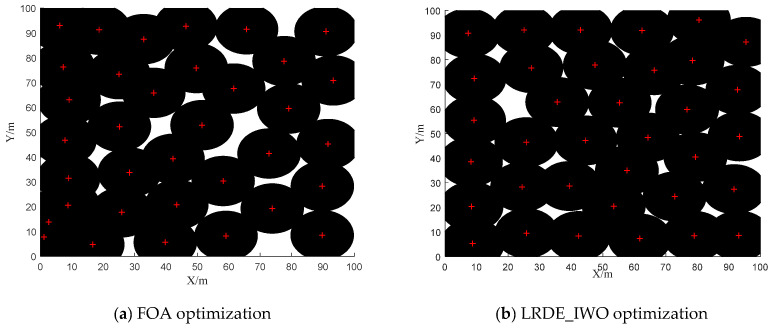
Node distribution optimized by FOA and LRDE_IWO.

The experimental parameters are set as follows: the monitored area is a two-dimensional plane of 100 m × 100 m, which is discretized into 101 × 101 grid points, a number of sensor nodes of N=35, a sensing radius of $R_{s}=10$ m, a communication radius of $R_{c}=20$ m and a maximum iteration of 300. Figure 8 shows the iteration curve and Figure 9 shows that the optimized node deployment diagram. Table 6 shows the comparison of the FOA and LRDE_IWO optimal coverage results. 

As shown in Table 6, the optimization results of LRDE_IWO are 2.73% higher than the FOA algorithm. From Figure 8, it can be seen that the algorithm converges faster and has a higher coverage accuracy than the FOA algorithm. Figure 9 shows that LRDE_IWO effectively reduces the coverage blind area. Experiments show on average 35 nodes are required for the FOA algorithm, if coverage rate reaches 90.82%, while Table 6 shows that when 35 nodes are deployed, the coverage rate of LRDE_IWO can reach 93.55%. The optimal distribution of the corresponding nodes is shown in Figure 9b.

Through many experiments, LRDE_IWO can achieve 90.82% coverage rate by deploying only 32 nodes in this area. It saves 8.5% sensor nodes compared with the FOA algorithm and makes the optimal distribution of nodes more even.

The simulation results show that the boundary coverage of LRDE_IWO is 91.64%, and that of FOA is 86.25% with 35 nodes deployed. This shows that the coverage of LRDE_IWO is wider than that of FOA, which can effectively reduce the impact of the coverage blind area.

##### (3) Compared with the Coverage Optimization Algorithm Based on IIWO

**Figure 10:**
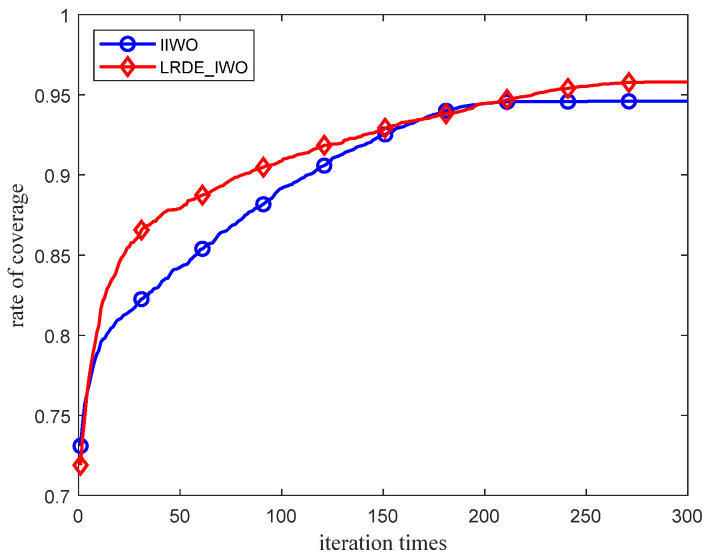
Coverage optimization iterative curve of IIWO and LRDE_IWO.

**Figure 11:**
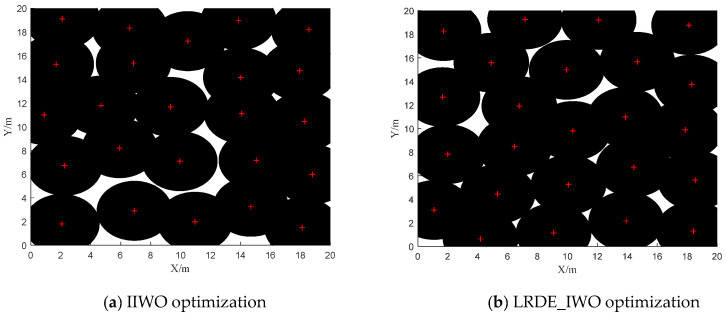
Node distribution optimized by IIWO and LRDE_IWO.

The experimental parameters are set as follows: the monitored area is a two-dimensional plane of 20 m × 20 m, which is discretized into 21 × 21 grid points, a number of sensor nodes of N=24, a sensing radius of $R_{s}=2.5$ m, a communication radius of $R_{c}=5$ m and run independently 20 times with 300 iterations each. Figure 10 shows the iteration curve of the IIWO and LRDE_IWO algorithms. Figure 11 shows the optimized node deployment, and Table 7 shows the comparison of the optimal coverage results of IIWO and LRDE_IWO. 

As can be seen from Table 7, after 20 node deployment optimizations by LRDE_IWO in this area, the average, best and worst coverage values obtained by LRDE_IWO are 1.19%, 1.36% and 1.81% higher than IIWO algorithm, respectively, and the standard deviation of the coverage is relatively smaller. This indicates that the optimum coverage of the LRDE_IWO algorithm can be more stable than 94.33%.

The experiments show that the LRDE_IWO can achieve a 94.79% coverage rate by deploying 23 sensor nodes in the area. It saves 4.1% sensor nodes compared with the IIWO algorithm.

The simulation results show that the average boundary coverage of LRDE_IWO is 94.01% and that of IIWO is 91.84%. This shows that the coverage effect of LRDE_IWO is better and more stable than that of IIWO, which is the key to reduce the coverage blind area.

## 7. Conclusions

Node deployment is an important issue in the research field of wireless sensor network. A hybrid strategy-based improved weed algorithm (LRDE_IWO) is proposed to optimize the coverage of sensor nodes based on the invading weed algorithm. The algorithm uses the normal distribution standard deviation of the tangent function as a new step search method for seeds. It effectively balances the global exploration and local development capabilities of the algorithm. Then, for enhancing population diversity, Levy flight or adaptive random walk strategies are introduced. Finally, differential evolution is applied to improve the problem that the algorithm is prone to fall into the local optimum. 

The LRDE_IWO algorithm is applied to WSN node deployment optimization. The simulation results show that its coverage is higher than that of IWO, DE_IWO, ALO, FOA and IIWO algorithms. Moreover, it improves the coverage, convergence speed and convergence accuracy to a large extent compared with other algorithms.

## Figures and Tables

**Table 1:** Experimental parameters.

Area	Perceived Radius	Communication Radius
20 × 20	2.5	5
50 × 50	5	10
100 × 100	7	14

**Table 2:** Comparison of the results in the 20 m × 20 m area and 24 nodes.

Algorithm	Mean cr	Best cr	Worst cr	Std
Based on IWO	92.46%	95.24%	90.02%	0.0114
Based on DE_IWO	93.73%	95.46%	90.93%	0.0099
Based on LRDE_IWO	95.65%	96.83%	94.78%	0.0061

**Table 3:** Comparison of the results in the 50 m × 50 m area and 35 nodes.

Algorithm	Mean cr	Best cr	Worst cr	Std
Based on IWO	90.40%	92.50%	87.47%	0.0138
Based on DE_IWO	92.16%	93.30%	88.67%	0.0128
Based on LRDE_IWO	93.99%	94.21%	92.21%	0.0061

**Table 4:** Comparison of the results in the 100 m × 100 m area and 80 nodes.

Algorithm	Mean cr	Best cr	Worst cr	Std
Based on IWO	90.87%	92.35%	90.15%	0.0088
Based on DE_IWO	92.34%	94.20%	90.80%	0.0094
Based on LRDE_IWO	96.43%	97.09%	95.49%	0.0051

**Table 5:** Comparison of the optimized coverage results between ALO and LRDE_IWO.

Algorithm	Mean cr	Best cr	Worst cr	Std
Based on ALO	93.11%	95.83%	91.93%	0.0107
Based on LRDE_IWO	97.21%	98.34%	96.43%	0.0052

**Table 6:** Comparison of the optimized coverage results between FOA and LRDE_IWO.

Algorithm	cr with 40 Nodes Deployed	cr with 35 Nodes Deployed
Based on FOA	95.19%	90.82%
Based on LRDE_IWO	97.29%	93.55%

**Table 7:** Comparison of the optimized coverage results of IIWO and LRDE_IWO.

Algorithm	Mean cr	Best cr	Worst cr	Std
Based on IIWO	94.60%	96.15%	92.52%	0.0097
Based on LRDE_IWO	95.79%	97.51%	94.33%	0.0078

## Data Availability

Not applicable.
